# 

**Published:** 1895-06-01

**Authors:** 


PRICE TWOPENCE.

No. 453, Vol. XVIII,—June 1st, 1895.

[Registered at the General Post Office as a Newspaper.

WITH EXTRA NURSING SUPPLEMENT

Per Annum, 10s. 6d., post free.

Monthly Parts, 6d.; post free, 8d.

Ditto per Annum, 8s. 6d., post free.

